# The System Justification Conundrum: Re-Examining the Cognitive Dissonance Basis for System Justification

**DOI:** 10.3389/fpsyg.2016.01889

**Published:** 2016-11-30

**Authors:** Chuma K. Owuamalam, Mark Rubin, Russell Spears

**Affiliations:** ^1^School of Psychology, University of Nottingham, Malaysia CampusSemenyih, Malaysia; ^2^School of Psychology, The University of NewcastleCallaghan, NSW, Australia; ^3^Department of Psychology, University of GroningenGroningen, Netherlands

**Keywords:** system justification, cognitive dissonance, system dependency, social identity, personal and group interests

In a landmark 1994 publication in the *British Journal of Social Psychology*, Jost and Banaji proposed the existence of a novel, fundamental system justification motive that drives social behaviors. More specifically, they proposed (a) that people have an epistemic need to support social hierarchies and societal systems, (b) that this *system justification* motive is inversely related to personal and group interests among members of low status groups, and (c) that it is stronger and more effective for people who are disadvantaged by societal systems than for those who are advantaged by them, especially when personal and group interests are weak. This system justification theory (SJT) has faced theoretical opposition from social identity researchers (e.g., Spears et al., 2001; Reicher, 2004; Rubin and Hewstone, 2004). In addition, evidence against the theory has recently accumulated from large scale cross-national studies (e.g., Brandt, 2013; Kelemen et al., 2014) and experimental studies (Trump and White, 2015; Owuamalam et al., 2016). In the present article, we re-examine the key cognitive dissonance assumptions for SJT's central proposition that support for unequal systems should be higher among members of disadvantaged groups than among members of advantaged groups when personal and group interests are weak.

## *Why* should members of disadvantaged groups be most likely to justify the system that disadvantages them?

At the heart of SJT is the idea that the motive to support or *justify* social hierarchies and systems operates separately from personal or group interests. Jost and Banaji (1994) were emphatic about this point, stating that: “system-justification does not…[operate] in the service of protecting the interests of the self or the group” (p. 10). However, operationally it is difficult (though not impossible) to distinguish personal, group, and system motives. One useful approach has been to focus on the responses of members of disadvantaged groups because, although SJT assumes that personal and group interests *reinforce* the system motive among people who belong to *advantaged* groups (e.g., European Americans), the theory also assumes that personal and group interests *conflict with* the system motive among people who belong to *disadvantaged* groups (e.g., Black/African Americans). Hence, the system justification that is shown by members of disadvantaged groups can be attributed entirely to the system justification motive rather than to personal and/or group motives, which would predict a lack of support for the system. Indeed, based on cognitive dissonance theory (Festinger, 1962), SJT proposes that members of disadvantaged groups will be *more likely* than their privileged counterparts to experience *cognitive dissonance*: That is, a conflict between the need to improve their disadvantaged position in the social hierarchy (i.e., personal/group interest) and the need to support the existing social order (the system motive). Consequently, SJT proposes that members of disadvantaged groups will be more motivated and more likely than members of advantaged groups to resolve this cognitive dissonance by embracing societal systems that disadvantage them.

## *When* should members of disadvantaged groups be most likely to justify the system?

Cognitive dissonance theory outlines a number of strategies that people may use to resolve the tension between their attitudinal preferences and a starkly opposing social reality. For example, people may adjust their attitudinal preferences so that they become compatible with reality and the status quo (i.e., social stasis). Alternatively, they may attempt to change reality so that it is in alignment with personal and collective preferences (i.e., social change). SJT assumes that people will be most likely to resolve their cognitive dissonance and associated uncertainties in the first way, by supporting social systems, when they feel that the system is stable and unchangeable (cf. Jost et al., 2012). SJT also proposes that this system justification should be most likely when personal and group interests are *weak*. As Jost et al. (2004) explained:

The strongest, most paradoxical form of the system justification hypothesis, which draws also on the logic of cognitive dissonance theory, is that members of disadvantaged groups would be even more likely than members of advantaged groups to support the status quo, *at least when personal and group interests are low in salience* [emphasis added] (p. 909).

The reason for this prediction is that personal and/or group motives may at times overwhelm and obscure the system motive and, consequently, the effects of the system motive are only likely to be apparent when personal and group motives are weak.

## An inconsistency between cognitive dissonance theory and SJT

There is an important problem with SJT's assumption that system justification should be most apparent when personal and group interests are weak. Cognitive dissonance theory predicts that people will experience a dilemma (or cognitive conflict) only when a belief or attitude that is *important* to them runs counter to reality. As Festinger (1962) explained:

The magnitude of the dissonance, of course, will also be affected by those variables that affect the importance of the cognitive elements involved in the dissonance. The more *important* [emphasis added] the elements, the greater will be the magnitude of the dissonance (pp. 179–180).

Hence, only people who have *strong* attitudinal preferences toward (or attachment to) their disadvantaged group identities should experience a significant cognitive conflict with a social reality that disadvantages their group. If people are *weakly* invested in their disadvantaged group identity, then it is unlikely that this weak attitudinal preference will be sufficient to cause a conflict with a disadvantageous social reality, with the implication that system justification should be less likely, not more likely. Subsequent refinements of cognitive dissonance theory have, if anything, only strengthened the claim that the key elements creating dissonance must be important, central and self-relevant. For example, Aronson (1994) stated that “…my own research has led me to conclude that dissonance effects may be limited to situations where our behavior violates our own self-concept…” (p. 231). Empirical evidence by Simon et al. (1995) also supports the moderating effect of the personal importance of the elements that are involved in cognitive dissonance effects (see also Smith and Mackie, 2007). Even Jost et al. (2003, p. 32) acknowledge this inconsistency but do not provide a reason for it.

Perhaps for this reason, recent revisions of SJT have suggested that system justification should be most likely to emerge when people are *dependent* on the system for some benefit, such as access to healthcare and education (Kay et al., 2009) or remunerations and salaries (van der Toorn et al., 2015). This *system dependence* is thought to increase the sense of cognitive dissonance and subsequent system justification. However, the distinction between personal/group interests and system dependence is open to question on both theoretical and empirical grounds. Theoretically, it is unclear why dependence on a social system (e.g., healthcare) would not be strongly related to (vested) personal and group interests associated with that system. Empirically, there are some open questions about the research that supports the distinction by contrasting system dependence and personal/group interests.

For example, Kay et al. (2009) exposed participants to an experimental manipulation of system dependence (see Table 1) and then examined the effect of this manipulation on Rosenberg's (1965) Self-Esteem Scale (their proxy for personal-interest), Luhtanen and Crocker's (1992) Collective Self-Esteem Scale (their proxy for group-interest), and a measure of system dependency. Consistent with predictions, Kay et al. found that their system dependence manipulation increased participants' system dependence but did not affect their personal or collective self-esteem. Based on this evidence, they concluded that system dependence is independent from personal and group interests. However, a closer look at Kay et al.'s (2009) measure of system dependency raises some doubts about this conclusion. First, Kay et al.'s two-item measure of system dependence appears to measure personal and group interests. The first item states: “The decisions and actions of the federal government *affect me personally* [emphasis added]” (p. 427). The second item: “*Individual Canadians success'* [emphasis added] depends on the government making good decisions” (p. 427) also refers to a potential mix of personal and group interests.

**Table 1:** **operationalization and manipulation of system dependency as put forward by Kay et al. (2009)**.

	**Operationalization of system dependency**	
	**Country dependency (p. 425)**	**University dependency (p. 425)**
Measurement of system dependency (two items, p. 427): “The decisions and actions of the federal government affect me personally,” and “Individual Canadians' success depends on the government making good decisions”	Many young people feel that the decision they make in terms of where to live is a very important one. In fact, recent surveys report that even at age 40, people still consider that their choice to live where they do was one of the most impactful decisions of their life. Indeed, sociological studies comparing the outcomes of residents of various countries show that there might be some truth to these perceptions. *In particular, it seems that the country you live in has enormously broad effects on your life and wellbeing. In terms of financial wellbeing, for instance, the taxes you pay, the job and investment opportunities made available to you and the general state of the economy are all to a large extent under the control of your country's government. But even in terms of social and personal wellbeing, the country you live in has substantial impacts: the quality of your social services (health and education), the leisure activities you have access to and time to pursue, even the likelihood that you will be happy with your eventual life-partner—all these aspects of your life are ones that are, at least according to these studies, to some degree dependent on the country you live in.*	Many new students feel that the decision they made to attend their particular university was a very important one. In fact, recent surveys of university alumni report even at age 40 that their choice of university was one of the most impactful decisions of their life. Indeed, sociological studies comparing the outcomes of students and alumni of various universities show that there might be some truth to these perceptions. *In particular, it seems that the university you attend has enormously broad effects on your life and wellbeing. In terms of financial wellbeing, for instance, the fees you pay and the job opportunities made available to you during and after graduation are all to a large extent under the control of your university. But even in terms of social and personal wellbeing, the university you attend has substantial impacts: the quality of your peers and professors, the extracurricular activities you have access to, the people you are likely to meet and befriend and even eventually settle down with—all these aspects of your life are ones that are, at least according to these studies, to some degree dependent on your university.*

Italics in the text above represents our emphasis and were not included in the original piece.

In addition, the operationalization of system dependency in Kay et al.'s (2009) study supports the view that personal interests are part and parcel of system dependency. As can be seen in Table 1, their operational manipulation of system dependency refers to the country or university that participants belonged to having: “enormously broad effects on your life and wellbeing…[and affecting] the taxes you pay, the job and investment opportunities made available to you…”. These examples all appear to be rooted in personal and/or group interests and, taking them into account, Kay et al.'s evidence seems to suggest that making personal and group interests salient increases people's personal and group interests as much as their system dependence. Thus, Kay et al.'s (2009) findings, as with van der Toorn et al.'s (2015), seem to be consistent with emerging evidence that system justification can go hand in hand with personal and group interests (e.g., Owuamalam et al., 2016, in press) rather than being in opposition to them or only coming to the fore when these interests are weak.

In short, researchers have yet to provide convincing evidence that system dependence is conceptually and empirically distinct from personal/group interests (i.e., either unrelated or inversely related). This makes it difficult to sustain the claim that people with weak personal or group interests, but high system dependence, will be subject to the most cognitive dissonance and thus the greatest system justification. More direct evidence for the precise dissonance mechanism mediating these effects is required.

## Can personal and group interests account for system justification?

We have discussed a key problem with SJT's system motive explanation for system justification amongst members of disadvantaged groups. Modifications to SJT that remove this motive render SJT's position conceptually similar to other mainstream accounts such as social identity theory (SIT: Tajfel and Turner, 1979). But this is not necessarily a bad thing. As Kay and Jost (2014, p. 146) acknowledged, “another, quite different approach is to distill a common denominator …and conclude that seemingly disparate theories are really all saying the same thing ….” A common denominator between SJT and its competition (e.g., SIT) could be that personal and group interests drive system justification even if accepting the status quo may seem costly to members of disadvantaged groups in the short term (e.g., Owuamalam et al., 2016).

## Conclusion

We examined the cognitive dissonance assumption underlying SJT (Jost and Banaji, 1994), and our analysis highlights a theoretical inconsistency between cognitive dissonance theory and SJT. SJT proposes that system justification should be most apparent among members of disadvantaged groups who have weak personal and group interests because (a) they have the largest discrepancy between their personal/group interests and their disadvantaged position and (b) the system motive is least likely to be overwhelmed by weak personal and/or group motives. In contrast, a straightforward interpretation of cognitive dissonance theory predicts that system justification should be strongest among members of disadvantaged groups when their personal and group interests are *strong*, not *weak*, because it is under these conditions that cognitive dissonance is at its greatest. Attempts to resolve this theoretical inconsistency with recourse to the additional concept of system dependency have not, in our opinion, been successful. Consequently, we recommend a revision of SJT that brings it more in line with the original predictions of cognitive dissonance theory. In this revision, personal and group interests may predict system justification amongst members of disadvantaged groups. Of course, if the system motive is dispensed with in favor of personal and group motives, then it is unclear what remains in SJT's account that makes it theoretically distinct from other theories of intergroup relations such as SIT. Nevertheless, SJT has provided an important platform for contemplating why members of disadvantaged groups justify the social system that disadvantages them. However, its cognitive dissonance explanation of the system justification effect is contestable. In our view, it is more parsimonious to explain system justification in terms of personal and group motives as emerging evidence now suggests (see Owuamalam et al., 2016).

## Author contributions

CO, MR, and RS contributed equally to the preparation of this opinion article.

### Conflict of interest statement

The authors declare that the research was conducted in the absence of any commercial or financial relationships that could be construed as a potential conflict of interest.
